# “It Should Have Been Given Sooner, and We Should Not Have to Fight for It”: A Mixed-Methods Study of the Experience of Diagnosis and Early Management of Cerebral Palsy

**DOI:** 10.3390/jcm10071398

**Published:** 2021-03-31

**Authors:** Sîan A Williams, Woroud Alzaher, Anna Mackey, Amy Hogan, Malcolm Battin, Alexandra Sorhage, N Susan Stott

**Affiliations:** 1 Curtin School of Allied Health, Curtin University, Perth 6845, Australia; 2 Department of Surgery, University of Auckland, Auckland 1023, New Zealand; s.stott@auckland.ac.nz; 3 New Zealand Cerebral Palsy Register, Starship Child Health, Auckland 1023, New Zealand; WoroudA@adhb.govt.nz (W.A.); AMackey@adhb.govt.nz (A.M.); ASorhage@adhb.govt.nz (A.S.); 4 The Cerebral Palsy Society of New Zealand, Auckland 1023, New Zealand; amy@cpsociety.org.nz; 5 Newborn Services, Starship Child Health, Auckland District Health Board, Auckland 1023, New Zealand; MalcolmB@adhb.govt.nz; 6 Department of Paediatric Orthopaedics, Starship Child Health, Auckland District Health Board, Auckland 1023, New Zealand

**Keywords:** early diagnosis, communication, parental support, early management

## Abstract

Listening to the family experience is integral to identifying areas of strength and for improvement in health service delivery around diagnosis and early management of cerebral palsy (CP). Families of children with a diagnosis of CP were invited to complete a purpose-developed electronic survey that included items around the timing of diagnosis, their experiences and satisfaction. It also allowed families to expand on their experiences through free text. Of the 57 families responding, 49% of children functioned at Gross Motor Function Classification System (GMFCS) levels I or II, 8% at GMFCS level III and 23% at GMFCS levels IV or V. 51% of participants were satisfied or very satisfied with the diagnosis experience, 18% were neutral about the experience and 31% were dissatisfied or very dissatisfied. Though the findings of this study may be subject to selection bias, perceived delays in the receipt of diagnosis of CP appeared common with 60% of participants indicating concerns about their child by <6 months of age but only 21% provided with a diagnosis of CP <6 months of age. Approximately 18% of families experienced a delay of more than 12 months. Thirty-four (61%) participants noted a delay between referrals to a service and receipt of service management/therapy. Common themes impacting on families’ experience in the diagnosis and health service delivery journey related to provision of information, and the style of communication, with both direct and ongoing communication styles common for greater family satisfaction. Overall, families desired the diagnosis experience to be informative and timely, with early follow up support and assistance with health sector navigation.

## 1. Introduction

Frequently referred to as the most common motor disability in childhood, cerebral palsy (CP) is a complex lifelong condition attributed to neurological impairment in the developing foetal or infant brain [1]. Heterogenous in causality and presentation (with varying degrees of functional severity), the lived experience of CP for an individual and their family is multifaceted, and shaped by personal and environmental factors [2], usually beginning within the health service provision setting. The receipt of the diagnosis of CP presents a family with one of the first significant moments in the management of the condition. Avieli & Band-Winterstein 2017 [3] provide a powerful description of the parental phase of ‘seeking extrinsic recognition of the pathology by diagnosis’, believed to play a central and crucial part in shaping parental role for years to come [3]. Despite the early acquisition of the neurological injury, a child will not typically receive a diagnosis of CP until they are between 8–24 months of age [4]. This delay can result in a loss of opportunity for targeted early intervention with the potential to shape developmental trajectories from childhood through to adulthood [5], and may expose families to additional uncertainty whilst they wait.

Compelling evidence of the accuracy of key clinical assessments tools now confirm that a diagnosis of CP, or identification that a child may be a high risk of CP, can be made before six months of age [5]. With an algorithm for clinical ‘options’ [5], the recommended combination use of Magnetic Resonance Imaging (MRI) [6], The Prechtl Qualitative Assessment of General Movements [7] and Hammersmith Infant Neurological Examination [8] for infants with a clinical history of risk factors are highly accurate for the prediction of CP (>97% [9]). Though the availability of accurate tools has now removed a key historical barrier to early diagnosis (or an interim diagnosis of ‘at risk of CP’), health professionals still may feel hesitant to provide families with such significant information so early. Yet, with the increasing evidence around the benefits of early intervention [10,11,12] and support for families, the barriers to early diagnosis continue to fall. The family/person centered approach championed in health service delivery [13], and strengthened shift towards a stronger inclusion of people with the lived experience in our approach to both health care and research reminds us again that we need to hear and acknowledge the family perspective when it comes to health care. Especially given that this childhood disability is a lifelong journey, and the valuable relationship between the family, child, and the medical profession.

Previous publications indicate that families are aware of the delay in diagnosis of their child [14,15,16], and reports significant dissatisfaction with the diagnostic process, often linked to the delay itself [15]. In addition to this, delays in diagnosis are associated with parental depression [15], increased parental stress [17], poor adaptive coping [15,17], and a developing mistrust for health care professionals [16]. Most families report early suspicions during the ‘wait and see’ period [15], and frustration either relating to the timing of diagnosis, or with failure to be given diagnostic information of any kind [18]. Therefore, the aim of this study was to survey the family experience surrounding diagnosis and early management of infants with CP in Aotearoa New Zealand. A secondary aim was to identify areas of strengths and areas for improvement in health service delivery around diagnosis and early management, from the family perspective.

## 2. Materials and Methods

### 2.1. Participants

Families of children who had received a diagnosis of CP in Aotearoa New Zealand (birth years 2008–2018) were invited to participate in the online survey (QualtricsXM) between October 2018 and November 2019. Families registered with the New Zealand Cerebral Palsy Register (NZCPR) and who had consented to being informed of research opportunities were notified of the survey initially by a mail out (https://nz.cpregister.com/ (accessed on 30 March 2021)), though we were unable to confirm receipt of mail out. The survey was also advertised digitally on the Facebook page for The CP Society of New Zealand (a member-based organization for people with CP and their families, https://cerebralpalsy.org.nz/ (accessed on 30 March 2021)), providing an anonymous link for families to follow directly to the online survey, and promoted further by word of mouth at consumer research days. Participants completing the survey acknowledged that they were a member of a family with a child with CP who had been born within the previous 10 years, and that they consented to participate (Ethical approval Health and Disability Ethics Committee: 18/NTB/169).

### 2.2. Instrument

Survey development was guided by Burns et al. [19]. Initial survey items were generated based on the study objectives (divided into three themes); (i) the family experience surrounding the diagnosis of CP, or identification of high risk, (ii) the family experience of early management, including referral, receipt and satisfaction of services and support, and (iii) area of strengths in current service delivery and areas for improvement in diagnosis and early management. In addition, it included participant demographic information. Survey questions were developed and refined with input from group sessions between the research investigators, external consultation with people who had a lived experience of CP, and through a review of the literature. Items were reduced to less than 25 items to minimize surveyor burden [20], and ‘display logic’ was included to customize the questions based on the respondents answers. The survey was, again, reviewed by the team to ensure it addressed the objectives, and following feedback refinements were made to improve clarity and reduce redundancy, and to prioritize the order of the survey questions according to the research objectives.

The survey initially opened at the participant information sheet and consent, followed by screening questions to ensure they met the study criteria and consented to participate. Investigator contact details were provided again at the end of the survey in case families felt distressed from answering the questions, the survey also provided a separate link to allow survey respondents the opportunity to provide additional anonymous feedback to the research team.

### 2.3. Data Analysis

Data were excluded if <30% of the survey (i.e., only screening questions) had been completed (*n* = 31 excluded). Data were collated to provide frequencies (and percentages) of responses. Free text response questions allowed participants to provide written responses to question, with responses collated using NVIVO12, and two authors completing a thematic analysis (SW, WA) to highlight primary themes. Text responses specifically corresponding to the question regarding satisfaction with how the diagnosis was delivered were categorized by participant response and linked to themes.

## 3. Results

### 3.1. Participant Demographics

A total of 57 families participated in the survey between Oct 2018 and Nov 2019, with majority of the responders being mothers (*n* = 50 mothers). Children were born between the year 2008 and 2018 (2008–2012 *n* = 33, 2013–2018: *n* = 24) in New Zealand (*n* = 2 were born in the United Kingdom and moved to New Zealand when the child was <6 months old), and majority (*n* = 45) received health care in the North Island of New Zealand (Table 1). Most (*n* = 53) families filled in details about their child’s functional classification of CP, with proportions of GMFCS levels matching that of interim outcomes from the NZCPR.

### 3.2. Family Experience of the Diagnosis

**Figure 1:**
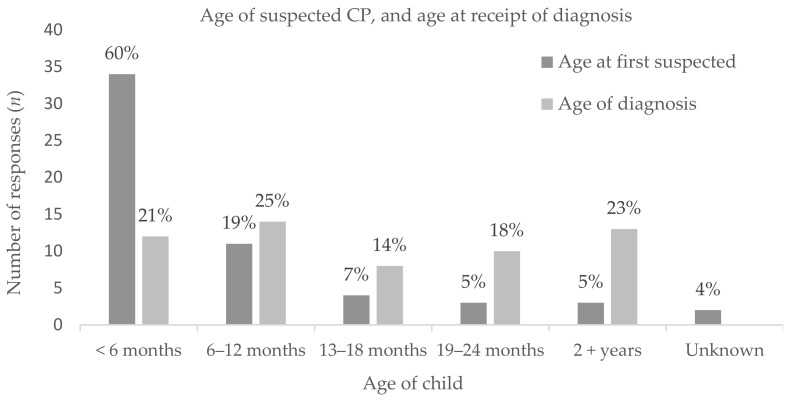
Age of child whereby families first suspected that their child may have CP (or a like condition), vs. the age the family was given a diagnosis of CP (Cerebral Palsy).

Families indicated at what age they first suspected/identified their child may have CP (or a like condition), and at what age their child then received their diagnosis (i.e., when the diagnosis itself was given, not an interim ‘at risk’). The majority (*n* = 34, 60%) suspected/identified when their child was under 6 months old, but only 21% (*n* = 12) were diagnosed under 6 months (Figure 1). When asked if they perceived a delay in the confirmation of diagnosis; No delay *n* = 7 (12%), 2 weeks-2 months *n* = 9 (16%), 3–6 months *n* = 9 (16%), 7–12 months *n* = 13 (23%), 13–24 months *n* = 8 (14%), 2.5 years+ *n* = 2 (3%), and Unsure *n* = 8 (14%).

General Paediatricians were the most reported health professional (*n* = 23, 41%) to be providing the diagnosis of CP (Table 2). Whether or not participants were provided with clear information about diagnostic tests, what CP is, what the future may involve and time to ask questions at the time of diagnosis and months soon after are outlined in Table 2. 51% (*n* = 29) of families indicated they were satisfied/very satisfied with how the diagnosis was delivered.

### 3.3. Themes of Expereinces

**Figure 2:**
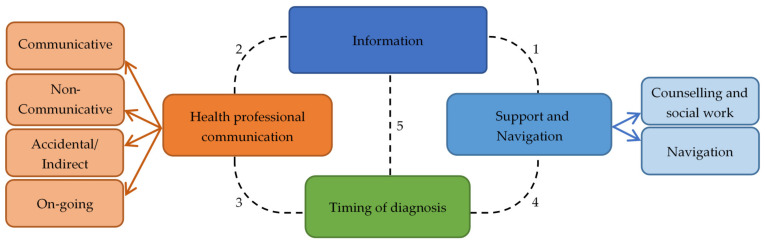
Main and sub-themes in open-ended responses to family survey. Dotted lines indicate links between themes: (**1**) Lack of information and support were linked themes, families without information noted feeling unsupported. (**2**) Responses describing ‘communicative’ health professionals frequently also described receiving information, linking the two themes. (**3**) Families describing an ‘on-going’ communication style from health professionals regarding diagnosis also described their diagnostic experience positively, though it remained lengthy in many cases. (**4**) Families describing extreme frustrations with delayed diagnosis also described feeling not listened to and not supported, linking the two themes. (**5**) Families who experienced a delayed diagnosis noted their lack of diagnostic information was harmful to accessing beneficial targeted therapy.

Figure 2 summarises the common themes identified by the free text responses, further elaborated below in text, with Table 3 outlining the number of times the themes (and sub themes) were discussed by families for the level of overall satisfaction the diagnosis process.

#### 3.3.1. Theme: Information Provided about Diagnosis and Outcomes

‘Information’ emerged as a higher order theme that linked all themes, including timely information about the diagnosis, the changing child needs over time, and information about social support. Families describing positive health practitioner interaction, either because they were communicative or provided an ‘on-going diagnosis’ (i.e., under surveillance for/at risk of CP) approach, noted they received vital information that either satisfied their search for answers or enabled them in seeking initial therapy. Similarly, for families caught in a lengthy diagnosis process, responses indicated a lack of information. Families with clinicians who were responsive to their changing information needs felt empowered and enabled in their caretaker role. Direct family request for significantly more information was a frequently suggested improvement to the health system (*n* = 19 responses, *n* = 15 families) including direct requests for a comprehensive and all inclusive ‘information pack’. A request for thorough information was closely linked with family priorities to maximise their child’s potential.

#### 3.3.2. Theme: Health Professional Communication

Family responses frequently referred to health practitioner communication of the diagnosis with varying experiences, some positive and others noting communication issues. Responses related to communication identified four communication styles: communicative, non-communicative, accidental or indirect communication, and on-going communication.

##### Sub Theme: Communicative

Seventeen responses (*n* = 17 responses, *n* = 14 participants) described communicative health professionals and noted they were responsive to the family needs in their communication. In this group, health practitioners’ communication was described as honest, informative, detailed, clear and the clinicians’ manner as caring and sensitive. Other important descriptors in family responses when communication went well was that health practitioners balanced honesty with being sensitive, and combined reassurance with a clear description of effects on development.

*“The paediatrician […] met with us in her office 2 days later and we talked for 2.5 h. She has a very holistic view of children’s development. She did NOT sugar-coat anything—she admitted, honestly, the potential problems we may face in the future.”*

Participant 29

*“We described the milestones that our child wasn’t hitting. The paediatrician was lovely, she didn’t interrupt or anything. Once we had finished speaking, she calmly explained she knew why that was, and explained that our child had cerebral palsy. She was very considerate of how we might react and took her time to explain to us in full what this will entail.”*

Participant 68

##### Sub Theme: Non-Communicative

Health practitioners were not always communicative about diagnostic information (*n* = 16 responses, *n* = 12 participants), which was commonly seen for families indicating a ‘Dissatisfied’ experience. Families noted a lack of conversation to introduce possible diagnoses and a lack of discussion around what CP was and how it may affect their child. One family noted their practitioner was too soft in their explanation of MRI findings and that the family was significantly misguided, highlighting the importance of clear communication.

*“We were referred to a Neuro-developmental therapist who came to our house to meet with us. She walked through our door and within the first minute stated, “ Oh yes she is a typical CP Baby”. That was how we were diagnosed basically! We did not get all the information about CP and what it meant at the time of diagnosis.”*

Participant 30

*“When we were told at birth about the brain damage on the MRI scan, the Doctor didn’t tell us clearly enough (she was too soft about it) and we both came away thinking it was possible our daughter would be OK. Someone had to re-explain to us a couple of days later that she would have lifelong disability and that the brain damage was not reversible.”*

Participant 52

##### Sub Theme: Accidental or Indirect Communication

Several families (*n* = 9 responses, *n* = 9 participants) noted they (accidentally) discovered a CP diagnosis for their child through health practitioner communication that assumed family pre-knowledge of it. Examples included the family copied into communication between health practitioners, appointment notes or a physical letter in the post mentioning the diagnosis. Families with this experience were extremely dissatisfied and shocked, and expressed disappointment and anger that an important diagnosis was not communicated.

*“Had raised concerns with nurses who visited as son was premature…. Was informed nothing to be concerned about. Raised concerns again with paediatrician at regular review. She noted our concerns but never told us what she was suspecting. We received a LETTER in the post which stated she suspected hemiplegia. To be informed about this is a letter was horrendous. I will never forget that day and not having a medical practitioner to talk to and would never wish receiving a diagnosis in the mail on anyone.”*

Participant 53

*“I thought it was a general paediatrician appointment. He said sorry that the results from MRI 8 months ago were incorrect and that PVL is the cause of her CP. Then he realized that I had no idea she even had CP.”*

Participant 39

*“Our daughter experienced a hypoxic event at birth, was diagnosed with HIE. We knew the CP diagnosis was coming but it was never formalized verbally to us—just added to appointment notes.”*

Participant 28

Eight participants (*n* = 8 responses) noted clinician’s indirect communication about a diagnosis, with clinicians mentioning ‘diplegia’, ‘muscle tone’ and ‘hemiplegia’ without identifying a root diagnosis. Families described learning from their physiotherapist that the root cause was CP.

*“I think being expected to understand the diagnosis though reading a letter from the outpatient clinic saying, “may be developing diplegia” and then having a physiotherapist a few months later explain that this means highly likely CP was not very good. Also, we [then] had to wait months before we saw the developmental paediatrician which caused anxiety as we wanted to be doing all we could for our son so he could have the best outcome.”*

Participant 5

##### Sub Theme: On-Going Communication

Several (*n* = 8) participants described an ongoing communication approach where health professionals shared information about possible differential diagnoses as it became known to them, most responses in this sub-theme were associated with a ‘very satisfied’ response to overall satisfaction with diagnosis. Families described being aware of the chances of CP as a diagnosis and that they were in an observation/surveillance period before it could be confirmed. Although families in this group were in an uncertain phase without a confirmed diagnosis, they noted their involvement in the diagnostic process as observers was beneficial. When these families received a formal diagnosis, they described the experience as ‘expected’ and ‘inevitable’ and highlighted that the crucial conversations they had during the ‘wait and see’ period enabled them to seek treatment to optimise their child’s neurodevelopment.

*“At 15 days old we were told he had 80-90% chance of having CP and that it would likely be severe based on an MRI. This was not a diagnosis as we were left with a wait and see. He was 2 years before the diagnosis was officially given but by then it was not a shock more an inevitability.”*

Participant 40

*“We knew we were likely dealing with cerebral palsy and the paediatrician had highlighted to us she was under observation for it. We officially recovered our diagnosis when we communicated to the Dr that we thought she had it and he agreed. It was a gentle approach enabling us to accept it when we were ready.”*

Participant 1

#### 3.3.3. Theme: Time to Diagnosis

Perceived delays in diagnosis and health practitioner resistance to formally diagnose were noted frequently by families (*n* = 19 responses, *n* = 13 participants). Families commented that health professionals missed too many opportunities to say CP was a possible diagnosis. For these families, the lack of diagnostic information to work with was damaging as they felt it delayed their access to support and targeted therapy during a time of neuroplasticity.

*“Paediatricians need to risk being wrong and give a diagnosis. The not knowing and wondering what else could be wrong is horrible.”*

Participant 26

*“There we so many opportunities for medical professionals to inform us that our son was high risk of having CP however this information was never passed on to us. […] I know doctors can’t look into the future, but giving a parent an idea in what to expect would be helpful. Your whole world turns upside down at diagnosis stage. […] He was in NICU [Neonatal Intensive Care Unit] 6 weeks and this was never mentioned.”*

Participant 53

Families described extreme experiences, such as repeatedly communicating their concerns over their child’s development without apparent clinician response or demanding a diagnosis. Families described being under considerable stress as they could not target therapy for their child. Responses had strong emotive descriptions including words such as ‘devastating’, ‘horrendous’, ‘nobody wanted to listen to us’, ‘had to fight for it’ and ‘your world turns upside down’.

*“If I could change one thing it would be earlier diagnosis. She was blue at birth! Delayed from the start, had we had CP explained from beginning and therapy with appropriate therapist she may have progressed faster. […] [The diagnosis] should have been given sooner and we shouldn’t have had to fight for it.”*

Participant 36

*“For 9 odd months my wife kept saying something was wrong, then when finally confirmed, the response was ‘we sometimes get it wrong, oh well’”*

Participant 37

*“Would like to have been referred earlier, very stressful feeling like you can’t do anything for your child, but you know something just isn’t right with them.”*

Participant 49

Possible reasons for a delayed diagnosis as reported by families were general reluctance to provide a formal diagnosis, clinician perception that providing a diagnosis would essentially stop involved health professionals looking for answers and the use of indirect communication that addressed the symptoms without mention of the root cause.

*“We asked for a label for our son’s conditions to given so we could access support services. It was clearly explained to us that it was given with caution as once given the medical professionals tend to stop looking for an answer.”*

Participant 33

*“It took almost 2years for a Dr to diagnosis, they just keep saying “why do you want that diagnosed she already has lots of diagnosis.” It wasn’t till a visiting paediatrician came to town and saw my daughter that they agreed my daughter has CP and put it down as a diagnosis.”*

Participant 22

Some families had a positive experience with an early diagnosis or were provided with a working/on-going diagnosis (*n* = 14 responses, *n* = 14 participants). This overlapped with the on-going communication style as families with a working diagnosis also had direct clinician communication about what was certain and what possible diagnoses were. Families highly valued having transparent information about what the clinician was observing for. The transparent open communication provided them with as much certainty as was possible. In comparison to emotive words above, responses describing an early or on-going diagnosis used words ‘expected’, ‘gentle approach’, ‘expected shock’, and a feeling of being ‘prepared’.

*“Having an initially warning of about 5 months that that might be what was going on. I felt like the idea was slowly broken in on us.”*

Participant 8

*“Luckily the Paediatrician understood the need early on for our daughter to have a confirmed diagnosis (even though she was very young) so we had certainty and could access services. He was also very direct and clear which I appreciated.”*

Participant 52

#### 3.3.4. Theme: Support and Information about the Navigation of the Health Service Journey

##### Sub Theme: Family Support—Counselling and Social Supports

Fifty-five (of 57) families in the survey responding to open text questions noting that that their child’s diagnosis had a [huge/significant] effect on their whānau (extended family). Families described financial stress, social isolation, and mental health impacts in association with their child’s diagnosis. Though families’ experiences widely varied, including families with and without social and financial support, families noted that it was a ‘sad’ and ‘stressful’ experience and used highly emotive words including ‘frustrating’, ‘hard’, life changing’, ‘traumatic’ and ‘devastating’. Families noted a lack of social support offered, including counselling.

*“After that we were left to sort it out ourselves once the referral to the [hospital] went through. We weren’t put in touch with a counsellor or social service to help us which in hindsight was very poor.”*

Participant 34

*“Support, counselling, where to go and talk to someone with experience. The other things we were offered at the time felt quite offensive. Instead of feeling like an advocate for my child I was seen as being defensive or demanding and hard to deal with.”*

Participant 2

*“Recognizing that it is not only the child with the diagnosis that is affected. The whole family is and every decision impacts ALL of those involved.”*

Participant 33

##### Sub Theme: Health System Navigation

Families found that the health system was, at times, difficult to navigate with a relatively high number of appointments, large health care team and multiple services involved (*n* = 19 responses, *n* = 17 participants). Families commented on the overwhelming administrative component in navigating a health system for a complex diagnosis, with no support or guidance in this process. Families frequently suggested a care coordinator, particularly as the system was noted to be not integrated and being connected with a family with a similar journey (*n* = 9 responses, *n* = 9 participants). Having a relatively easy and integrated health system was valued by the respondents as it allowed them to focus on their child’s health care.

*“Early on I would have liked more therapy options as well as more opportunities for networking with other families with children with similar needs.”*

Participant 40

*“A knowledgeable point-of-contact for parents of kids with complex diagnoses. There are just so many appointments in those early years, it is incredibly stressful and hard to keep up.”*

Participant 52

### 3.4. Early Management

Thirty-four (61%) participants said there was a delay between referrals to a service and receipt of service management/therapy. Twenty-four participants responded in text about the length of delay, which varied from 2 months to 2 + years; 2–6 months delay *n* = 12, 6–12 months *n* = 6, 12–18 months *n* = 4, >2 years *n* = 2. Forty-five (80%) participants responding saying that they had sought information/additional therapy external to your health care provider).

## 4. Discussion

Despite advances in evidence for the use of accurate assessment diagnostic tools for diagnosing children with, or identifying children who are at high risk of CP [5], findings from the current study indicate that families in Aotearoa New Zealand do perceive a delay in the diagnosis process, though nearly half reported to have received their diagnosis before 12 months of age. This study captured a range of experiences, about half of the families were satisfied with the experience but saw opportunity for improvement in the clarity of information on the diagnostic assessments, on what CP is, and what the future may look like for their child. Information was an overarching desire relating to the family experience, linked also to health professional communication styles, timing of the diagnosis process, and navigation and support in the health service journey. Providing timely discussions around diagnosis (or a possible diagnosis of CP) allows for the potential for early targeted intervention and surveillance, but also provides families the opportunity to seek support, receiving counselling, and plan for their child’s future.

In 2013, Shevell and Shevell [21] discussed common findings of parent perceived delays in the disclosure of the diagnosis of CP identified from studies spanning 1990 [14], 2000 [15], and 2010 [16]. Despite numerous calls to minimize delays in the diagnosis of CP [22], families are still reporting frustration with delays a decade later. Having an early diagnosis or being involved in an on-going (i.e., interim at risk of CP) diagnosis was associated with higher satisfaction levels with the overall diagnosis experience. A visual time shift delay between initial suspicions and subsequent diagnosis of 6 months was observed in the current study, similar to the average 7 months interval identified by Dagenais et al., 2006 [17]. Comparatively, 60% of participants in our study indicated suspicions of CP (or like condition) under 6 months of age but only 21% of families were provided a diagnosis of CP when their child was under 6 months of age, with almost 20% of families noting a delay in diagnosis of more than 12 months. Though changes are underway, the current use of previously discussed diagnostic tools remain low in New Zealand [23], as such, clinicians may feel hesitant to provide an early diagnosis in favor of waiting to see how the child’s development progresses with age. However, parents are observant, with most holding suspicions before being told [15,24]. Cottrell and Summers (1990) [24] suggest that parents want to be told as soon as disability is suspected. Health professionals may “hedge” to avoid inaccuracy, but, by doing so, risk generating doubt and frustration [18], and anger [15] during the delay. Baird et al., 2000 [15] reported a consistent trend for parents of children diagnosed later to be more dissatisfied, with parents indicating that they would have liked to have been warned of possible adverse outcomes from their child’s prematurity. Although it is not always feasible for health professionals to provide an ‘early’ diagnosis of CP, the use of diagnostic tools to identify risk and an interim diagnosis of high risk of CP, as per best practice recommendations [5], is a strong message that is echoed within the current study.

The age-old adage ‘Information is key’ was also applicable to the responses around communication styles. Positive experiences centered around being honest, informative, and timely, suggesting also that families desire the experience to be compassionate, with follow up support and opportunity for questions. Some of the less positive experiences saw frustrations with delay and concerning accidental ‘discoveries’ of the diagnosis (via referral letters or case notes) and details being vague or brief. Suspicions of diagnoses being withheld, or concerns being dismissed were also apparent. Physicians can be reluctant to share uncertainties around diagnoses and outcomes. However, Guttmann et al. 2018 [18] found that, in a study of NICU graduates, regardless of the strategy used to identify infants at high risk for CP, honest conversations surrounding the limitations of prognostication are important for preserving trust and recommended physicians consider presenting parents with a range of potential outcomes for children with motor disability. This type of ongoing communication led to high parent satisfaction in this study, indicating that parent’s value being part of the diagnostic journey, if a confirmed diagnosis is not yet possible. Conversely, ambiguity around information with misleading or partial information can lead to parents “groping in the dark”, searching for answers, as commented by Avieli and Band-Winterstein [3]. Parental preparedness for the diagnosis is also key and sharing information about expected developmental skills for age, suggestions for promoting skills, and specific time frames for follow-up evaluation can enhance parental understanding [25]. Similarly, health professional attributes such as approachability, understanding, sympathy, and hopeful realism is valued by parents [17,25].

Parents commented on challenges relating to a lack of post-diagnosis support such as counselling, and information or guidance that might help them navigation the systems. An absence of clarity in health management pathways was also noted recently by NZ clinicians [23], with the majority indicating they would be in favor of a more consensus approach to the management of CP. Over recent years several websites, infographics and facts sheets have been developed to provide reputable and accessible information for families and people with CP and health practitioners around the world. Steps should be taken to adapt such information (where appropriate) for different cultural contexts and country specific health care systems and encourage widespread awareness of these resources across the CP community.

### Limitations

Generous responses provided within the free-text responses of the surveys provided ample content to draw from for a thematic qualitative analysis, though individual interviews or focus groups may have allowed for more detailed and free flowing insights. A response rate was not able to be determined for this study as the survey was distributed predominantly via anonymous links on social media, and we were unable to confirm how many suitable participants may have been notified of the study via the NZCPR. The generalizability of our findings is thus reduced as parents who were content with the diagnostic experience may have elected not to answer the survey. Nevertheless, the responses show that there is a vast range in the family experiences, and within that range includes a group of families for whom the diagnostic experience is less than optimal. The generalizability may also be limited geographically, ethnically, and towards a skewed sample (parents choosing to join a non-profit society) favoring families actively seeking community support. It could be speculated that children with less severe functional impairments (i.e., lower GMFCS levels) may be more inclined to experience delays in diagnosis, as such, our sample may have captured those who may have been more likely to fall within that category. However, the spread of participants across all GMFCS and MACS levels appears representative of the CP population. The study did not seek information about the aetiology of CP, and thus cannot comment on associations between family experience, the casual pathway of acquisition of CP or the pathway of health service delivery. Finally, with the intent to optimize relevance of experience to more current practice in New Zealand, a deliberate decision was made to limit the recency of the ‘experience’ to the previous 10 years (i.e., the age of the child >10 years). Though this remained an inclusion criterion, the research team were contacted by families eager to share detailed recollections of their experiences (not included for analysis), highlighting the long-lasting significance of the diagnosis experience.

## 5. Conclusions

This paper sought to highlight the strengths and factors relating to positive experiences, such that clinicians may feel better prepared to support families through their journey. No health professional set out to provide a less-than- satisfactory experience for families, yet in the face of complex medical conditions, competing priorities, and concern of limited referral services, the experience of diagnosis may not always be optimal for families. Health professionals need to walk the tightrope of being honest about challenging outcomes and diagnoses, yet also hopeful and supportive [26]. Families of children with CP are perceiving delays in their child’s diagnosis process, despite good clinician awareness of diagnostic tools, which may be owing to the indications that the current combination use of diagnostic tools appear to be low [23]. This study reiterates other findings in strong support of provision of a diagnosis (or an interim diagnosis of at risk of/under surveillance for CP), recommending that clinicians follow previously establish guidance for disclosure of diagnosis [15], and highlights that information, communication [17,27,28] and timing is key. There may be an art to providing the right timing/pace of information delivery for families, and there may well be no perfect formula to suit all, but capturing the voice of families is an important step in shaping our evolving approach to the diagnosis and early management of CP. It is important to reflect that the provision of a diagnosis (or interim ‘at risk’) does not preclude a hope and strength-based approach to management that includes family centered discussions and goal settings for the child’s future. Indeed, earlier conversations with families may facilitate improved direction for understanding the child’s individual needs, both in the immediate term and in the future. Findings provide insight for frequent positive experiences in health care such as, compassion, honesty, supportive that we should also be celebrated. Practical solutions that draw on the positive and negative aspects of the family experience may serve to improve health service provision for families of children with CP.

## Figures and Tables

**Table 1:** Family and child characteristics.

					Total *n* (%)	
**Responder** (*n* = 57)						
Mother					50 (88%)	
Father					2 (3.5%)	
Mother and Father					2 (3.5%)	
Grandparent					3 (5%)	
**Number of children in the family** (*n* = 57)						
1 child (with CP)					15 (26%)	
2 children					29 (51%)	
3 children					9 (16%)	
4 children					4 (7%)	
**Prioritised ethnicity of child with CP** (*n* = 57)						
New Zealand European					42 (74%)	
Māori					11 (19%)	
Pacific					1 (<2%)	
Asian					1 (<2%)	
Prefer not to answer					1 (<2%)	
			**Childs birthplace**		**Receipt of health services**	
North Island (NZ)			44 (77%)		47 (82.5%)	
South Island (NZ)			11 (19.5%)		10 (17.5%)	
UK			2 (3.5%)			
**Topographical classification** (*n* = 57)						
			**Hemiplegic**	**Diplegic**	**Quadriplegic**	**Unknown**
			21 (37%)	16 (28%)	16 (28%)	4 (7%)
**Functional classification**						
	**I**	**II**	**III**	**IV**	**V**	**Unknown**
GMFCS (*n* = 53)	12 (23%)	16 (30%)	4 (7.5%)	7 (13%)	6 (11.5%)	8 (15%)
MACS (*n* = 48)	4 (8.5%)	14 (29%)	3 (6%)	5 (10.5%)	5 (10.5%)	17 (35.5%)

CP: Cerebral Palsy, GMFCS: Gross Motor Function Classification System, MACS: Manual Ability Classification System. N.B. descriptions of each classification (and external links) were provided within the survey for participants for reference.

**Table 2:** Family experience of diagnosis, including who (i.e., which health professional) provided the diagnosis, information provided at the time of diagnosis and the months, and a rating of satisfaction with the delivery. A total (*n*) and percentage is provided for each response.

**Health professional providing the diagnosis** (*n* = 56)					
General Paediatrician	23 (41%)	Neonatologist & General Paediatrician			1 (1%)
Pediatric Neurologist	12 (21%)	Neonatal follow-up/Pediatric fellow			1 (1%)
Developmental Paediatrician	9 (16%)	General Practitioner			1 (1%)
Neonatologist	2 (4%)	Physiotherapist			1 (1%)
Occupational Therapist	3 (5%)	PICU Consultant			1 (1%)
Pediatric Rehabilitation Consultant	2 (4%)				
**At the time of diagnosis and the months following, where you given:**		**Yes**	**Yes, not enough**	**No**	**I do not recall**
Clear information about what the diagnostic tests/assessments were for?		24 (43%)	13 (23%)	14 (25%)	5 (9%)
Clear information about what cerebral palsy is?		21 (38%)	12 (21%)	21 (38%)	2 (4%)
Clear information about what the future may involve for your child?		17 (30%)	17 (30%)	22 (39%)	0 (0%)
Time to ask questions and clarify your understanding?		27 (48%)	15 (27%)	11 (20%)	3 (3%)
**Family satisfaction with diagnosis experience** (*n* = 57)					
Very satisfied		13 (23%)			
Satisfied		16 (28%)			
Neutral		10 (18%)			
Dissatisfied		11 (19%)			
Extremely Dissatisfied		7 (12%)			

**Table 3:** Number of free text responses containing content relating to key themes, stratified by family satisfaction of diagnosis experience.

Theme		Extremely/Satisfied (*n*)	Neutral (*n*)	Extremely/Dissatisfied (*n*)
Health Professional Communication	Communicative	12	1	1
	On-going	7	1	
	Non-communicative		4	8
	Accidental/Indirect		3	6
Time to diagnosis	Early/ongoing	8	3	3
	Delayed/withheld	4	1	8
Support	Navigation	8	4	5
	Family & social support	28	10	16

## Data Availability

The data presented in this study are available on request from the corresponding author. The data are not publicly available due to ethical reasons, public sharing of data was not specifically consented for by participants.
